# Generalized additive model integrating multi-source data for short-term influenza forecasting in Shenzhen, China (2023–2025)

**DOI:** 10.3389/fpubh.2026.1811040

**Published:** 2026-04-30

**Authors:** Xing Li, Qiuying Lv, Jianpeng Xiao, Zhigao Chen, Aiping Deng, Zuhua Rong, Huiyang Sun, Shu Xiao, Shisong Fang

**Affiliations:** 1 Guangdong Provincial Institute of Public Health, Guangdong Provincial Center for Disease Control and Prevention, Guangzhou, Guangdong, China; 2 Shenzhen Center for Disease Control and Prevention, Shenzhen, Guangdong, China; 3 Guangdong Provincial Center for Disease Control and Prevention, Guangzhou, Guangdong, China; 4 School of Public Health, University of South China, Hengyang, Hunan, China; 5 Department of Public Health and Preventive Medicine, School of Medicine, Jinan University, Guangzhou, Guangdong, China

**Keywords:** forecasting, generalized additive model, influenza, multisource data, SARIMAX

## Abstract

**Background:**

The COVID-19 pandemic has reshaped the global epidemiology of respiratory infectious diseases, posing new challenges for influenza forecasting. Existing studies are often limited by reliance on single data sources, poor interpretability, or failure to account for nonlinear relationships among variables, which restricts their ability to balance prediction accuracy and practical utility for public health decision-making. This study aimed to develop and validate a multisource data-integrated generalized additive model (GAM) to forecast influenza activity in Shenzhen, China.

**Methods:**

Using surveillance and auxiliary data from 2023 to 2025, we developed GAM models incorporating local and Hong Kong influenza surveillance, cross-boundary mobility metric, meteorological factors, and Baidu Search Index data. The predictive performance of the GAM was compared with Seasonal Autoregressive Integrated Moving Average with Exogenous Variables (SARIMAX) model. Model accuracy was evaluated using root mean square error (RMSE), mean absolute percentage error (MAPE), and R^2^.

**Results:**

The multisource data-driven GAM exhibited high predictive accuracy across short-term forecasting horizons. For 1-week ahead forecasts, the model achieved an R^2^ of 0.85 (95% CI: 0.74–0.92). Notably, performance remained robust for 2- and 3-week forecasts, with R^2^ values of 0.80 (95% CI: 0.69–0.87) and 0.74 (95% CI: 0.62–0.83), respectively. The GAM demonstrated superior overall performance compared with SARIMAX.

**Conclusion:**

The multisource data-integrated GAM provides robust and stable influenza forecasts for Shenzhen up to 3 weeks in advance. This approach provides a valuable tool to support cross-boundary public health collaboration between Hong Kong and Shenzhen, and might serve as a reference for the development of broader regional public health strategies in future research.

## 1 Introduction

According to the World Health Organization (WHO), influenza is a globally significant respiratory infectious disease, responsible for an estimated one billion annual cases and 3–5 million cases of severe illness (1), thereby imposing a substantial disease burden (2). This burden shows marked heterogeneity across regions, with southern Chinese provinces becoming a focus of concern due to their atypical influenza epidemic patterns (3). The epidemiology of influenza is characterized by distinct regional variations, particularly evident in the diverse seasonal dynamics across different climates and geographical zones (4). Compared with northern China, which mainly exhibits winter–spring epidemics, southern China (including Guangdong, Fujian, and other southeastern provinces) has year-round influenza activity and multiple epidemic peaks (4). The COVID-19 pandemic has fundamentally reshaped the global landscape of respiratory infectious diseases (5), presenting unprecedented challenges for influenza forecasting. On one hand, co-circulation of multiple pathogens has become the new norm, with influenza viruses frequently alternating or overlapping with respiratory syncytial virus, adenovirus, and other respiratory pathogens (6–8), significantly interfering with traditional influenza surveillance indicators such as influenza-like illness percentage (ILI%) and complicating the accurate assessment of epidemic intensity. On the other hand, non-pharmaceutical interventions implemented during the pandemic, including mask-wearing and social distancing (9, 10), while effectively suppressing COVID-19 transmission, have profoundly altered population exposure levels and herd immunity backgrounds for influenza viruses, leading to a reconfiguration of seasonal influenza patterns.

As a major port city in southern China adjacent to Hong Kong, Shenzhen features high population density, frequent cross-regional mobility, and a humid subtropical climate with minimal seasonal variation. The confluence of these factors shapes a distinct epidemiological pattern for influenza, characterized by sustained year-round transmission and the regular occurrence of 1–2 epidemic peaks annually (11). Notably, Shenzhen has witnessed a substantial resurgence in influenza activity subsequent to the COVID-19 pandemic. Epidemiologically, the reported weekly incidence has surpassed 10,000 cases after 2023, markedly exceeding the pre-pandemic peak levels. Seasonally, the 2022–2023 influenza season exhibited a notably delayed winter peak with heightened intensity compared with historical trends, whereas the 2023–2024 season commenced its winter epidemic phase earlier than usual (12). Additionally, a distinct epidemic peak emerged during the autumn and winter of the 2024–2025 season. Collectively, these evolving epidemiological features—characterized by increased transmission intensity and shifted seasonal dynamics—pose ongoing challenges to influenza prevention and control in Shenzhen. Consequently, prediction models that rely solely on pre-pandemic historical data are no longer sufficient in this new epidemiological context. Therefore, elucidating the novel epidemiological features of influenza in post-COVID-19 megacities like Shenzhen and constructing adaptive prediction models represents a critical research need in public health.

The advancement of big data technologies has propelled researchers to explore non-traditional data sources to enhance predictive capabilities. For instance, individuals often search for related health information through search engines before and after developing influenza-like symptoms, and such behavioral data can be captured in real-time through platforms like Baidu Index, forming potential sentinel signals (13, 14). Meteorological factors (such as temperature, atmospheric pressure, wind speed, and humidity) have been extensively documented to influence influenza risk (15, 16). Furthermore, given the high frequency of cross-boundary mobility between Shenzhen and Hong Kong, influenza activity trends in Hong Kong may provide an imported early warning signal for Shenzhen, offering valuable predictive insights. However, the effects of these external drivers are not immediate and involve both time lags and nonlinearities. Traditional linear models struggle to fully capture these dynamics, making the systematic identification of effective predictors and determination of their optimal lag structures a key methodological challenge.

Existing predictive models often suffer from limitations such as reliance on single data source (17), poor interpretability (e.g., “black-box” machine learning models) (18), or failure to account for nonlinear relationships among variables (19, 20). These shortcomings hinder their ability to balance predictive accuracy with the interpretability required for public health decision-making. In contrast, generalized additive models (GAMs) offer a flexible semi-parametric framework capable of capturing nonlinear relationships, controlling for long-term and seasonal trends, and providing intuitive partial-effect interpretations (21). By maintaining high predictive performance while ensuring model transparency, GAMs align with the dual demands of scientific rigor and practical applicability in public health research.

To address these challenges, this study leverages a GAM framework to analyze weekly influenza positive rate data from Shenzhen (2023–2025). We aim to systematically evaluate the synergistic contribution of multisource heterogeneous data to influenza prediction in the post-COVID-19 era, thereby providing scientific evidence to inform precise influenza prevention and control strategies for densely populated megacities in southern China.

## 2 Methods

### 2.1 Study area and period

This study was conducted in Shenzhen, a high-density metropolis in southern China and a core city within the Guangdong-Hong Kong-Macao Greater Bay Area. Adjacent to Hong Kong, the city experiences substantial cross-boundary population mobility. The local subtropical monsoon climate contributes to its warm and humid conditions. The analysis utilized data spanning from Week 7 of 2023 to Week 50 of 2025, a period selected to encompass complete influenza epidemic cycles and capture distinct seasonal transmission patterns.

### 2.2 Data sources

#### 2.2.1 Shenzhen influenza surveillance data

The weekly influenza positive rate of specimens collected from ILI cases (influenza positive rate), and ILI% were obtained from the China Influenza Surveillance Information System, administered by the Shenzhen Center for Disease Control and Prevention (SZCDC). The current surveillance network in Shenzhen comprises 16 sentinel hospitals and 11 network laboratories. In each sentinel hospital, trained clinicians collected 10–20 respiratory specimens from patients meeting the influenza-like illness (ILI) case definition (body temperature ≥ 38 °C, with cough or sore throat) weekly. Specimens were transported via a cold chain to designated network laboratories within 24 h and maintained at 4 °C. Subsequently, these specimens underwent influenza virus nucleic acid detection using real-time polymerase chain reaction (22). Each network laboratory reported the weekly totals of tested ILI specimens and influenza-positive specimens to the SZCDC. The weekly influenza positive rate was calculated as the number of positive specimens divided by the total number of tested specimens.

The weekly ILI% was defined as the proportion of weekly ILI cases among the total outpatient and emergency visits across all sentinel hospitals in Shenzhen. Data on the dominant influenza strain were obtained from the weekly reports of SZCDC laboratory surveillance network. The dominant epidemic strain for a given week was determined through subtype composition analysis of influenza-positive specimens collected that week; the subtype (A/H1N1, A/H3N2, or B/Victoria) that accounted for the highest proportion among all positive specimens was designated as the dominant strain for that week.

#### 2.2.2 Hong Kong influenza surveillance data

Influenza surveillance data for Hong Kong were extracted from the official statistical datasets released by the Department of Health, Hong Kong Special Administrative Region. The dataset encompassed the following key indicators: the ILI emergency department consultation rate (%), the ILI consultation rate from sentinel general out-patient clinics (GOPCs, per 1,000 consultations), the ILI consultation rate from sentinel private medical practitioners (PMPs, per 1,000 consultations), the influenza A/B positive rate (%) in respiratory specimens, and the influenza admission rate in public hospitals with principal diagnosis of influenza (per 10,000 population).

These indicators are integral components of Hong Kong’s comprehensive influenza surveillance system. The system operates year-round and leverages a sentinel network that includes both public and private healthcare providers, ensuring robust community-level monitoring of influenza activity.

#### 2.2.3 Cross-boundary mobility data

The weekly data on inbound passenger volume from Hong Kong to Shenzhen were sourced from the official statistics of the Hong Kong Special Administrative Region Government Immigration Department.

#### 2.2.4 Baidu search index data

Baidu Search Index data, which aggregates search volume from both desktop and mobile platforms, were obtained from the Baidu Index official platform.^1^ The dataset was constructed using a set of influenza-related keywords, encompassing terms related to the disease itself, associated symptoms, and treatment options, as informed by previous literature (17). The following Baidu Search Index terms were used: influenza; myalgia, nasal congestion, fever, rhinorrhea, headache, sore throat, cough; oseltamivir, Tamiflu, and Xofluza. These keywords were selected to capture shifts in public awareness and concern regarding influenza.

#### 2.2.5 Meteorological data

Meteorological data, including weekly mean temperature, maximum temperature, minimum temperature, relative humidity, atmospheric pressure, sunshine duration, rainfall and wind speed were obtained from the daily monitoring records of the China Meteorological Administration. These daily records were subsequently aggregated to weekly resolution to align with the temporal scale of the influenza positive rate data.

#### 2.2.6 Temporal covariates

Temporal covariates were constructed using the calendar information spanning the study period to capture systematic time-based patterns in influenza transmission. These included a continuous time trend variable, which quantified the sequential progression of weeks to capture long-term or seasonal trends, and the count of public holidays within each week, which accounted for potential changes in social contact patterns during holiday periods.

### 2.3 Predictive model analysis

#### 2.3.1 Model and variable selection

GAM was selected as the primary analytical approach for its capacity to accommodate complex nonlinear relationships between predictors and the influenza positive rate, while preserving the interpretability of individual variable effects.

We implemented a multi-stage variable selection procedure to identify predictor variables that balanced predictive performance, model parsimony, and epidemiological interpretability.

First, a composite score equally weighting Spearman’s correlation and mutual information with the influenza positive rate was calculated. Variables scoring above the 20th percentile were retained. Second, to address multicollinearity while preserving domain-specific information, hierarchical clustering was applied. Before clustering, variables measuring closely related constructs from the same data source were manually grouped. All Hong Kong influenza surveillance variables listed in Section 2.2.2 were predefined as a single group to ensure they competed within the same cluster. The variable with the highest composite score was then selected from each cluster. Finally, pairwise Spearman correlations were examined; for any pair with r higher than 0.8, the variable with lower predictive importance was excluded, yielding the final set for GAM modeling.

Based on the biological characteristics of influenza, we incorporated three distinct lag windows (1, 2, and 3 weeks) into separate modeling frameworks.

#### 2.3.2 GAM model construction

As the outcome variable (influenza positive rate) is a proportion bounded between 0 and 1, and exploratory data analysis confirmed that its distribution was consistent with an under-dispersed Beta distribution, we employed a Beta regression GAM with a logit link function. The model formula is as follows:


$$\begin{array}{l} \log(\frac{\mu }{1-\mu })=\alpha +s(Z_{t1},\mathrm{df})+s(Z_{t2},\mathrm{df})+s(Z_{t3},\mathrm{df})\\ +\ldots \ldots +s(Z_{\mathrm{tn}},\mathrm{df})+s(\text{positive}\_\mathrm{rat}e_{\mathrm{lag}},\mathrm{df})+s(\text{time},7)+\text{holiday} \end{array}$$

Where, *μ* is the mean value of influenza test positive rate, ranging from (0,1); *α* is the intercept term; s is the nonparametric smoothing spline function; df represents degrees of freedom; Z_t1_-Z_tn_: predictor variables included after the multi-stage screening in section 2.3; positive_rate_lag_: lagged influenza positive rate; time and holiday represent long-term time trend and holiday effect, respectively.

#### 2.3.3 Rolling window prediction framework and performance evaluation

A rolling-window forecasting framework was adopted to systematically assess the model’s short-term predictive performance across different horizons. A fixed training window of 78 consecutive weeks (covering approximately 1.5 annual influenza cycles) was used to capture both seasonal and interannual variations while avoiding overfitting to a single season. This window length was selected because influenza seasonality in Shenzhen is not consistent across years, with substantial variation in the timing and magnitude of annual epidemic peaks. A window of only 1 full year may introduce confounding bias and unstable model estimation, as it would rely on a single highly variable seasonal pattern. In contrast, the 78-week window can adequately capture both seasonal and interannual variations of influenza activity, while ensuring sufficient sample size for stable model fitting and avoiding overfitting to a single season.

The training window was advanced weekly, with a rolling step of 1 week and three predictive horizons (1, 2, and 3 weeks ahead) assessed throughout the forecasting process. In each iteration, the model was trained on data within the current fixed window and then applied to predict the influenza positive rate at the predefined horizons. Specifically, in the initial iteration, the training dataset included weekly data from Week 7 of 2023 to Week 31 of 2024, which was used to develop predictive models for estimating the influenza-positive rate in Weeks 32, 33, and 34 of 2024 (corresponding to 1-, 2-, and 3-week-ahead predictions, respectively). In the subsequent iteration, the training window was expanded by incorporating data from Week 32 of 2024, and the updated dataset was used to generate 1-, 2-, and 3-week-ahead predictions for Weeks 33, 34, and 35 of 2024, respectively. This sequential window-updating process was repeated iteratively until predictions for the influenza positive rate in Week 50 of 2025 were completed. Such a design ensures a rigorous and consistent evaluation of the model’s generalizability across different lead times under real-world temporal dynamics of influenza epidemics.

Model performance was evaluated by Root Mean Square Error (RMSE), Mean Absolute Percentage Error (MAPE) and Coefficient of Determination (R^2^). RMSE quantifies the absolute deviation between predicted and observed values, with a range of [0, +∞); smaller values indicate better prediction accuracy. MAPE represents the relative prediction error in percentage terms, with a range of [0, +∞); lower values reflect better model fitting (23). R^2^ indicates the proportion of variance in the influenza positive rate explained by the model, with a range of [0, 1]; values closer to 1 suggest higher explanatory power. The total RMSE, MAPE, and R^2^ were the overall values calculated by merging the prediction data of all rolling windows, and their 95% confidence intervals (CI) were obtained through 1,000 Bootstrap samples.

#### 2.3.4 Model comparison

The predictive performance of the GAM was evaluated against a Seasonal Autoregressive Integrated Moving Average with Exogenous Variables (SARIMAX) model. SARIMAX was chosen for its strength in capturing seasonal patterns and the influence of external variables. The SARIMAX model shared the same exogenous predictors as the GAM, and its parameters were optimized by minimizing the Akaike Information Criterion (AIC). Within a unified rolling-window framework, both models generated 1-to-3-week-ahead forecasts, which were rigorously evaluated using RMSE, MAPE, R^2^, and Bootstrap 95% CI.

#### 2.3.5 Sensitivity analysis

To evaluate the robustness of the model, sensitivity analyses were conducted as follows: (1) the degrees of freedom for the time trend smooth term were set to 6 and 8; (2) the smoothing parameter estimation method was switched from restricted maximum likelihood (REML) to generalized cross-validation (GCV); (3) the rolling training window length was adjusted to 28 and 52 weeks.

All sensitivity models followed the same procedures as the primary analysis. Statistical analysis and visualization were performed in the R programming environment (version 4.3.1). The main packages included mgcv for GAM, forecast for SARIMAX, and ggplot2 for data visualization, along with other affiliated packages.

## 3 Results

### 3.1 Descriptive results

From Week 7 of 2023 to Week 50 of 2025, the weekly influenza positive rate in Shenzhen averaged 28.43% with a standard deviation (SD) of 20.06%, ranging from 0% to 76.47% (Table 1). The weekly ILI% exhibited a mean of 5.28% (SD:1.85%), with a range of 2.20% to 10.37% during the same period. Among the circulating subtypes, A(H3N2) was predominant, detected in 67 weeks (45.27%), followed by A(H1N1) in 64 weeks (43.24%), and B(Victoria) in 17 weeks (11.49%). Four distinct epidemic peaks were observed in early 2023, December 2023–January 2024, early 2025, and October–December 2025 (Supplementary Figure 1), underscoring the marked seasonal and fluctuating pattern of influenza activity.

**Table 1:** Descriptive statistics of daily influenza incidence and key predictor variables in Shenzhen, China.

Variable		Median (IQR) or Mean ± SD	Range	Weeks (%)
Influenza surveillance indicators in Shenzhen	Weekly Influenza positive rate (%)	28.43 ± 20.06	0.00–76.47	—
	Weekly ILI%	5.28 ± 1.85	2.20–10.37	—
	Predominant circulating subtype: A (H1N1)	—	—	64 (43.24%)
	Predominant circulating subtype: A (H3N2)	—	—	67 (45.27%)
	Predominant circulating subtype: B (Victoria)	—	—	17 (11.49%)
Influenza surveillance indicators in Hong Kong	ILI emergency department consultation rate (%)	152.37 ± 28.06	95.23–218.03	—
	Sentinel GOPC ILI consultation rate (per 1,000 consultations)	5.49 (3.99–8.43)	0.87–18.00	—
Sentinel PMP ILI consultation rate (per 1,000 consultations)	Sentinel PMP ILI consultation rate (per 1,000 consultations)	40.04 (33.82–46.44)	10.91–83.93	—
	Influenza A/B positive rate (%) in respiratory specimens	0.06 (0.02–0.09)	0–0.18	—
	Influenza admission rate in public hospitals with principal diagnosis of influenza (per 10,000 people)	0.33 (0.11–0.52)	0.01–1.21	—
Inbound passenger volume from Hong Kong to Shenzhen		1,771,753 ± 351,322	921,845–2,679,025	—
Baidu Search Index		13,078 ± 5,067	6,550–33,473	—
Meteorological variables	Weekly mean temperature (°C)	24.17 ± 4.71	10.47–30.21	—
	Weekly max temperature (°C)	30.52 ± 3.93	16.80–36.60	—
	Weekly min temperature (°C)	19.31 ± 5.81	3.90–26.70	—
	Weekly total sunshine duration (h)	58.02 ± 14.84	18.11–86.81	—
	Weekly total rainfall (mm)	9.05 (0.30–62.20)	0–302.10	—
	Weekly mean atmospheric pressure (hPa)	1005.19 ± 6.10	992.31–1019.73	—
	Weekly mean wind speed (m/s)	1.68 ± 0.35	1.10–3.03	—

In Hong Kong, the weekly ILI emergency department attendance rate had a mean of 152.37% (SD: 28.06%), while the median weekly influenza A/B positive rate was 6.00% (interquartile range, IQR: 2.00%–9.00%). For cross-boundary travel indicators, the weekly inbound passenger volume from Hong Kong to Shenzhen averaged 1,771,753 person-trips (SD: 351322; range: 921,845–2,679,025). The mean weekly Baidu Search Index for influenza-related searches was 13,078 (range: 6550–33,473). Regarding meteorological factors, the mean weekly temperature was 24.17 °C (SD: 4.71 °C), with values ranging from 10.47 °C to 30.21 °C. The mean weekly wind speed and atmospheric pressure were 1.68 m/s (SD: 0.35 m/s) and 1005.19 hPa (SD: 6.10 hPa), respectively.

### 3.2 Main results

The multi-stage variable selection process refined the initial set of 16 variables to a final combination of predictors, including Hong Kong’s influenza A/B positive rate (%) in respiratory specimens, ILI%, Baidu Search Index, atmospheric pressure, and the number of inbound travelers from Hong Kong to Shenzhen. The selection process is presented in Supplementary Figure 2.

Dose–response curves (Supplementary Figure 3) revealed the Hong Kong influenza A/B positive rate peaked in effect at 8%–10%. Atmospheric pressure and Baidu Search Index showed a U-shaped effect centered at approximately 1,005 hPa and 20,000, respectively. ILI% was associated with progressively stronger negative effects with increasing values. The number of inbound travelers from Hong Kong to Shenzhen showed no clear non-linear association with the weekly influenza positive rate.

**Figure 1:**
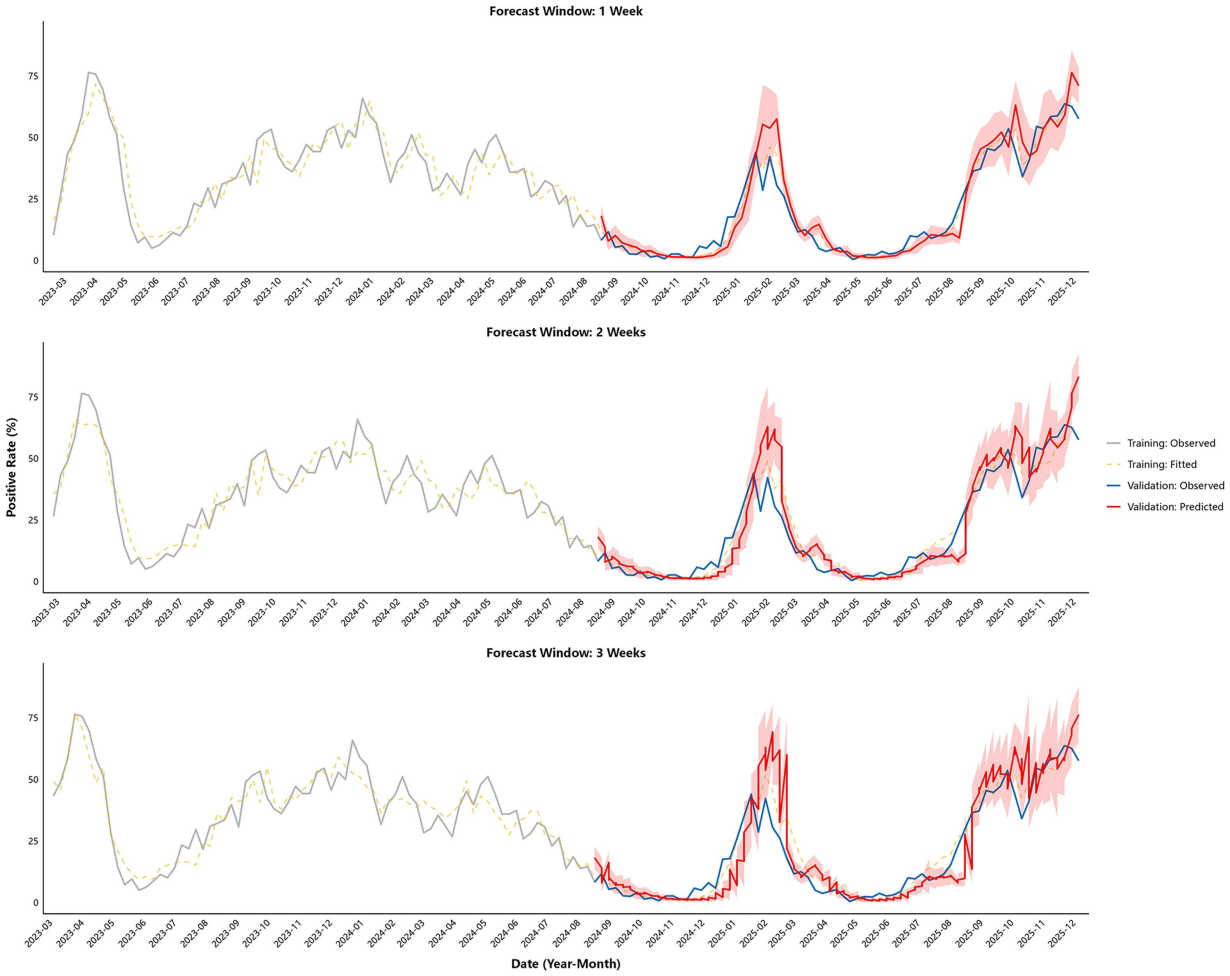
The fitting and predictive performance of the GAM model across different horizons.

The fitting and predictive performance of the model across different horizons are summarized in Table 2 and Figure 1. For the 1-week ahead horizon, the training set yielded an RMSE of 5.75 (95% CI: 4.94–6.59), a MAPE of 25.56% (95% CI: 20.63–31.17%), and an R^2^ of 0.91 (95% CI: 0.88–0.94). The corresponding validation set metrics were an RMSE of 7.45 (95% CI: 5.39–9.38), a MAPE of 45.82% (95% CI: 34.53–59.17%), and an R^2^ of 0.85 (95% CI: 0.74–0.92). For the 2-week horizon, the training set RMSE was 6.04 (95% CI: 5.30–6.81), MAPE was 27.74% (95% CI: 22.50–32.96%), and R^2^ was 0.90 (95% CI: 0.87–0.93). In the validation set, the RMSE increased to 8.66 (95% CI: 7.04–10.28), the MAPE to 51.17% (95% CI: 42.33–61.44%), and the R^2^ decreased to 0.80 (95% CI: 0.69–0.87). At the 3-week ahead horizon, the training set maintained strong performance with an RMSE of 5.67 (95% CI: 4.98–6.34), a MAPE of 32.96% (95% CI: 23.85–46.89%), and an R^2^ of 0.91 (95% CI: 0.89–0.94). The validation set performance further changed, showing an RMSE of 9.83 (95% CI: 8.20–11.47), a MAPE of 56.92% (95% CI: 48.62–66.48%), and an R^2^ of 0.74 (95% CI: 0.62–0.83).

**Table 2:** Rolling-window forecasting performance of GAM models across different prediction horizons.

Prediction horizon (weeks)	Training set			Validation set		
	RMSE	MAPE (%)	*R* ^2^	RMSE	MAPE	*R* ^2^
1	5.75 (4.94–6.59)	25.56 (20.63–31.17)	0.91 (0.88–0.94)	7.45 (5.39–9.38)	45.82 (34.53–59.17)	0.85 (0.74–0.92)
2	6.04 (5.30–6.81)	27.74 (22.50–32.96)	0.90 (0.87–0.93)	8.66 (7.04–10.28)	51.17 (42.33–61.44)	0.80 (0.69–0.87)
3	5.67 (4.98–6.34)	32.96 (23.85–46.89)	0.91 (0.89–0.94)	9.83 (8.20–11.47)	56.92 (48.62–66.48)	0.74 (0.62–0.83)

RMSE, root mean square error; MAPE, mean absolute percentage error (0.1 was added to the denominator to avoid division by zero); R^2^, coefficient of determination; Smaller RMSE and MAPE values and R^2^ closer to 1 indicate better model prediction performance.

### 3.3 Model comparison

The GAM model demonstrated superior predictive performance compared with the SARIMAX model across all prediction horizons (Tables 2, 3; Supplementary Figure 4). For the 1-week ahead prediction, GAM achieved a validation set R^2^ of 0.85 (95% CI: 0.74–0.92) versus 0.77 (95% CI: 0.65–0.88) for SARIMAX, with corresponding RMSE values of 7.45 (95% CI: 5.39–9.38) and 10.32 (95% CI: 7.06–13.48), respectively. The performance advantage was maintained at longer prediction horizons, with GAM consistently showing higher R^2^ values and lower RMSE and MAPE in both training and validation sets.

**Table 3:** Rolling-window forecasting performance of SARIMAX model across different prediction horizons.

Prediction horizon (weeks)	Training set			Validation set		
	RMSE	MAPE (%)	*R* ^2^	RMSE	MAPE	*R* ^2^
1	6.47 (6.31, 6.63)	31.14 (29.98, 32.47)	0.87 (0.87, 0.88)	10.32 (7.06, 13.48)	52.02 (38.05, 73.58)	0.77 (0.65, 0.88)
2	6.39 (6.25, 6.53)	32.18 (30.78, 33.67)	0.87 (0.87, 0.88)	10.87 (8.65, 12.89)	53.58 (41.77, 68.67)	0.75 (0.68, 0.83)
3	6.11 (5.96, 6.24)	32.70 (31.31, 34.22)	0.88 (0.88, 0.89)	11.17 (9.35, 13.03)	55.49 (45.09, 67.00)	0.73 (0.66, 0.80)

RMSE, root mean square error; MAPE, mean absolute percentage error (0.1 was added to the denominator to avoid division by zero), R^2^, coefficient of determination; Smaller RMSE and MAPE values and R^2^ closer to 1 indicate better model prediction performance.

### 3.4 Sensitivity analyses

Sensitivity analyses assessed the impact of training window length and model parameters on predictive performance (Supplementary Table 1). A monotonic improvement in predictive accuracy, as measured by *R*^2^, was observed with increasing training window length. The 28-week window yielded the lowest performance (*R*^2^ = 0.49–0.66), while the 52-week window, which aligns with the annual influenza cycle, showed acceptable performance (*R*^2^ = 0.58–0.78). The primary 78-week model achieved the optimal performance (*R*^2^ = 0.74–0.85). Furthermore, variations in the degrees of freedom for smooth terms (comparing df = 6 and 8) and the smoothing parameter estimation method (REML vs. GCV) resulted in no significant differences in model performance metrics (RMSE, MAPE, or *R*^2^) (Table 3).

## 4 Discussion

Based on data from 2023 to 2025, this study developed a GAM to forecast the influenza positive rate in Shenzhen. By integrating multi-source data, including local and Hong Kong influenza surveillance data, cross-boundary mobility metric, meteorological factors, and internet search indices, the model achieved robust performance across 1- to 3-week forecasting horizons, with R^2^ values of 0.85, 0.80, and 0.74, respectively.

A growing body of research has been conducted to forecast influenza activity. Unlike the suboptimal performance of GAM reported by Zhang et al. (24) in a multi-city study in China (R^2^ as low as −0.39 in Guangdong), our model, which incorporated Shenzhen-specific epidemiological characteristics and cross-boundary surveillance data, achieved significantly improved accuracy. This underscores the importance of developing location-specific forecasting frameworks. Furthermore, our GAM compared favorably against other advanced techniques. While Zhang et al. (24) noted that Autoregressive Moving Average-Generalized Autoregressive Conditional Heteroskedasticity (ARMA-GARCH) model and the Gated Recurrent Unit (GRU) models attained maximum R^2^ values of only 0.62 and 0.35, respectively, in Guangdong, our model outperformed these in short-term predictions. Recent studies applying machine learning methods, including Random Forest, eXtreme Gradient Boosting (XGBoost), and various deep learning architectures, for 1-week influenza forecasts have reported promising results (17, 25). For instance, research in a Hubei city documented R^2^ values of up to 0.84 for GRU and 0.79 for Long Short-Term Memory (LSTM) models. However, these investigations were typically limited to short-term (1-week) predictions and did not evaluate model performance over longer forecasting horizons. In addition, the multi-output Least Absolute Shrinkage and Selection Operator (LASSO) model developed by Shen et al. (13) showed a high R^2^ of 0.88 for 1-week predictions but declined sharply to 0.73 in the 2-week forecast. By contrast, our GAM maintained relatively stable performance over the 2- to 3-week horizon, suggesting its potential utility for public health planning.

Parameter configuration analysis revealed that the 78-week rolling window yielded superior forecasting performance across all horizons compared with the 28- and 52-week windows within the GAM framework. This advantage can be attributed to three principal factors: (1) enhanced stability in estimating nonparametric smooth terms due to a larger sample size, which reduced sensitivity to random noise; (2) inclusion of complete annual cycles and inter-annual transitions, enabling the model to represent the epidemiologically critical pathway from low-season viral circulation to the following seasonal outbreak—a dynamic missed by shorter windows; and (3) a favorable balance where the informational gain from more data surpassed the noise introduced by older observations, with no evidence of overfitting indicated by synchronous validation set improvements. Consequently, the conventional 52-week window, despite covering a full influenza year, proved less accurate for medium-term forecasts as it could not reflect these cross-annual dynamics.

Several features enhance the practical relevance of our study. First, by integrating surveillance data from Hong Kong and real-time cross-boundary mobility metrics, we enhanced the model’s specificity for Shenzhen’s unique epidemiological profile as a tightly connected metropolis with Hong Kong. This approach provides a replicable framework for similar border regions and facilitates cross-jurisdictional public health coordination. Second, the model’s ability to generate accurate 1- to 3-week forecasts offers actionable insights for public health preparedness, including resource allocation and vaccination campaigns. Third, the use of laboratory-confirmed influenza positive rates as the outcome variable improved specificity by reducing confounding from non-influenza respiratory pathogens—a notable advantage over ILI indicators, which are prone to nonspecific symptoms and reporting delays. Additionally, the inherent interpretability of GAM aids in identifying key drivers of influenza transmission, supporting evidence-based intervention planning.

Unlike machine learning models that only provide predictive accuracy without clear interpretability, GAM enables explicit quantification and visualization of associations between key factors and influenza positive rates, which is critical for translating research findings into practical public health strategies. Specifically, GAM’s interpretable nonlinear dose–response relationships (Supplementary Figure 3) offer targeted intervention guidance: preventing influenza transmission in Hong Kong may help reduce the risk in Shenzhen, while atmospheric pressure exhibits a U-shaped protective effect near 1,005 hPa, suggesting that alerts should be issued during extreme pressure conditions. Overall, GAM’s interpretability bridges epidemiological research and public health practice, facilitates evidence-based policy-making, and helps improve intervention effectiveness and reduce the influenza burden.

However, this study has several limitations. First, the nearly three-year study period, while covering the immediate post-pandemic phase, may not encapsulate long-term influenza dynamics. Second, while the present study accounts for cross-boundary travel between Hong Kong and Shenzhen, mobility data between Shenzhen and other mainland Chinese cities (including age-specific mobility for children) were not available for inclusion. The exclusion of this domestic travel data constitutes a limitation, as intra-mainland movement is a primary driver of influenza importation and local transmission in the region. Consequently, the current model may not fully capture the complete spectrum of population movement influences, which could affect the generalizability of our findings. Third, although the Baidu Search Index serves as a proxy for public awareness, it lacks the specificity to differentiate between preventive searches and symptom-driven queries and is susceptible to media-driven noise, potentially introducing confounding effects. Moreover, weekly influenza vaccination coverage data for the 2023–2025 period were not publicly available and thus could not be included in the models. Vaccination is a key factor influencing influenza transmission dynamics, and the lack of temporally resolved coverage data may introduce unmeasured confounding. However, it should be noted that Shenzhen has provided free influenza vaccination to priority groups since 2016 for adults aged ≥60 years and since 2019 for primary and secondary school students. The inability to adjust for time-varying vaccination coverage in our models is a notable constraint that should be considered when interpreting the findings. In addition, a key methodological limitation of this study lies in the scope of the comparative analysis. While benchmarking the proposed model against a linear SARIMAX framework demonstrates the advantages of explicitly modeling nonlinear relationships, this comparison alone does not provide a comprehensive assessment of its performance relative to state-of-the-art nonlinear predictive methods widely adopted in contemporary forecasting research, such as tree-based ensembles (e.g., Random Forest, XGBoost) or recurrent neural networks (e.g., LSTM). The absence of such comparisons may limit the generalizability of conclusions regarding the model’s relative competitiveness. Finally, as the model was trained and validated primarily in Shenzhen, its generalizability to cities with different climatic conditions or patterns of population mobility requires further evaluation.

Future research should aim to integrate additional variables—such as vaccination coverage, detailed human mobility trajectories, and intervention policies—over extended periods to improve model robustness across diverse epidemic scenarios. Moreover, future studies (e.g., Random Forest, LSTM, XGBoost) should conduct rigorous benchmarking against a suite of established nonlinear models under consistent input and validation protocols, thereby enabling a more robust and nuanced evaluation of predictive capability and practical utility. Finally, multi-center validation in cities such as Guangzhou, Zhuhai, and Hong Kong, along with integration into public health decision-support systems, will be critical steps toward translating these findings into practical tools for public health response.

## 5 Conclusion

In conclusion, this study developed a GAM model to forecast influenza positive rates that integrates multi-source data in Shenzhen. The model demonstrated robust and stable predictive performance across 1- to 3-week ahead horizons. These findings provide a scientifically grounded predictive tool for cross-boundary public health collaboration between Hong Kong and Shenzhen, and may provide a useful reference for the development of broader regional public health strategies in future investigations.

## Data Availability

The raw data supporting the conclusions of this article will be made available by the authors, without undue reservation.

## Footnotes

1
https://index.baidu.com

## Supplementary material

SUPPLEMENTARY FIGURE 1 Time series of influenza and key predictor variables in Shenzhen, China.

SUPPLEMENTARY FIGURE 2 The flow diagram of variable selection.

SUPPLEMENTARY FIGURE 3 Dose-response relationship curves of key preditors and influenza positive rate in Shenzhen.

SUPPLEMENTARY FIGURE 4 The fitting and predictive performance of the SARIMAX model across different horizons.
