# Supplementary material for: Generalized additive model integrating multi-source data for short-term influenza forecasting in Shenzhen, China (2023–2025)
Source: Front Public Health. 2026 Apr 30;14:1811040. doi: 10.3389/fpubh.2026.1811040 (PMC13173675; doi:10.3389/fpubh.2026.1811040)
Supplement: Supplementary file 1 [file Table_1.DOCX]

### ****Supplementary Table 1. Prediction performance of GAM models across different**** training window length and model parameters

| **Strategy** | **Prediction Horizon(weeks)** | **RMSE** | **MAPE(%)** | **R²** |
| --- | --- | --- | --- | --- |
| df=6 | 1 | 7.54(5.69–9.44) | 52.41(37.54–71.98) | 0.85(0.74–0.92) |
|  | 2 | 8.64(7.09–10.16) | 58.83(45.53–76.51) | 0.80(0.70–0.87) |
|  | 3 | 9.85(8.23–11.57) | 65.76(53.39–81.44) | 0.73(0.62–0.82) |
| df=8 | 1 | 7.17(5.04–9.07) | 44.41(33.34–57.37) | 0.86(0.75–0.94) |
|  | 2 | 8.19(6.63–9.62) | 48.70(40.11–58.89) | 0.82(0.72–0.89) |
|  | 3 | 9.52(8.07–10.99) | 54.94(46.92–63.98) | 0.75(0.65–0.83) |
| GCV | 1 | 7.45(5.39–9.38) | 45.82(34.53–59.17) | 0.85(0.74–0.92) |
|  | 2 | 8.66(7.04–10.28) | 51.17(42.33–61.44) | 0.80(0.69–0.87) |
|  | 3 | 9.83(8.20–11.47) | 56.92(48.62–66.48) | 0.74(0.62–0.83) |
| Window=28 | 1 | 11.21(9.02–13.16) | 35.96(29.79–42.19) | 0.66(0.52–0.79) |
|  | 2 | 12.67(11.02–14.12) | 39.92(34.54–45.12) | 0.57(0.44–0.68) |
|  | 3 | 13.71(12.11–15.20) | 44.62(40.03–49.38) | 0.49(0.36–0.61) |
| Window=52 | 1 | 8.67(6.66–10.81) | 37.52(29.79–46.61) | 0.78(0.62–0.87) |
|  | 2 | 10.19(8.37–12.08) | 43.75(35.77–52.90) | 0.68(0.54–0.80) |
|  | 3 | 11.71(9.82–13.52) | 51.83(44.09–60.64) | 0.58(0.42–0.71) |

Note: RMSE=root mean square error, MAPE=mean absolute percentage error (0.1 was added to the denominator to avoid division by zero), R²=coefficient of determination, AIC=Akaike information criterion; Smaller RMSE and MAPE values and R² closer to 1 indicate better model prediction performance; Smaller AIC value indicates better model fit.
