# Supplementary figures and images for: Generalized additive model integrating multi-source data for short-term influenza forecasting in Shenzhen, China (2023–2025)
Source: Front Public Health. 2026 Apr 30;14:1811040. doi: 10.3389/fpubh.2026.1811040 (PMC13173675; doi:10.3389/fpubh.2026.1811040)

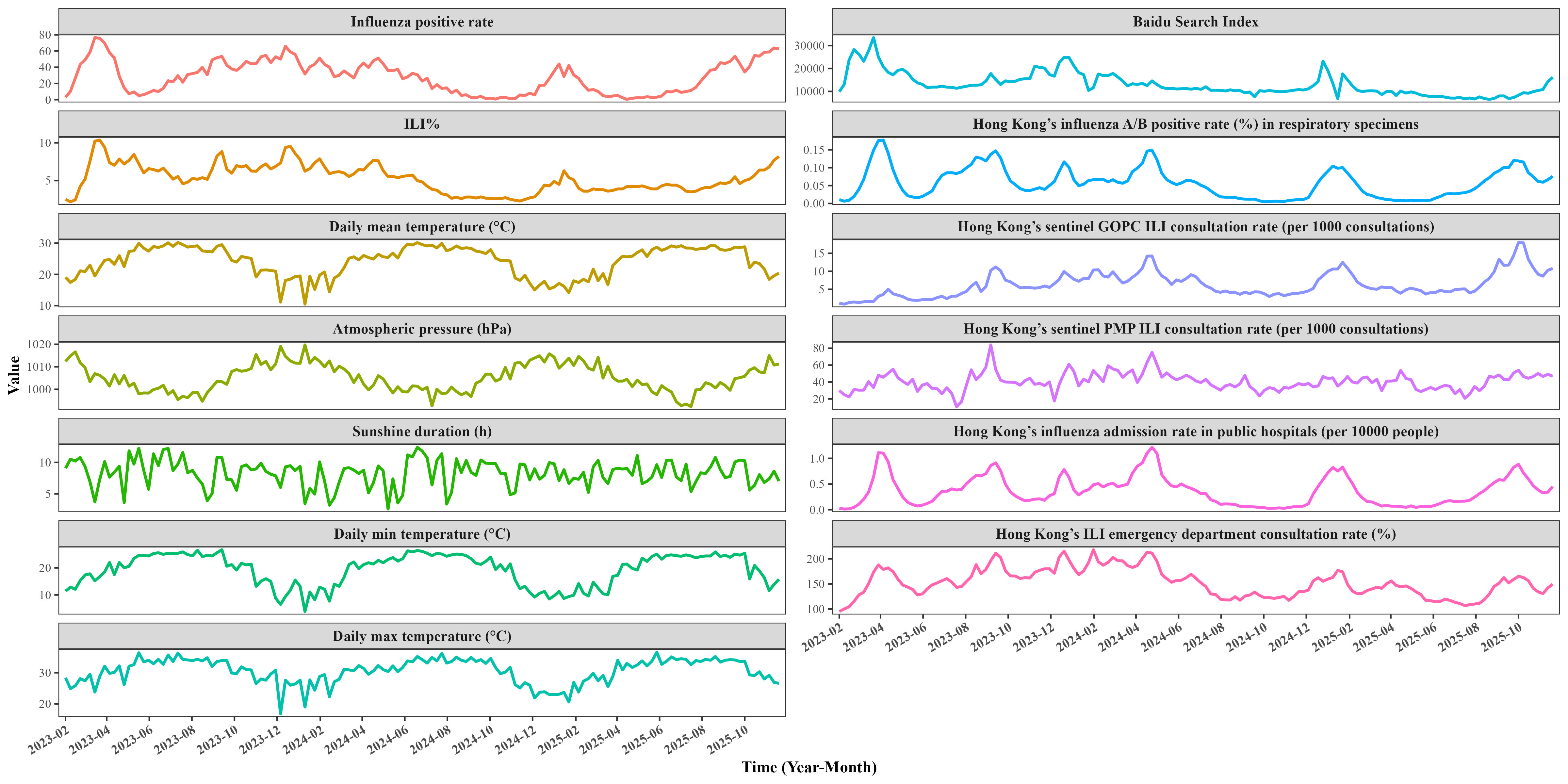

Supplement: SUPPLEMENTARY FIGURE 1 — Time series of influenza and key predictor variables in Shenzhen, China. [file Image_1.JPEG]

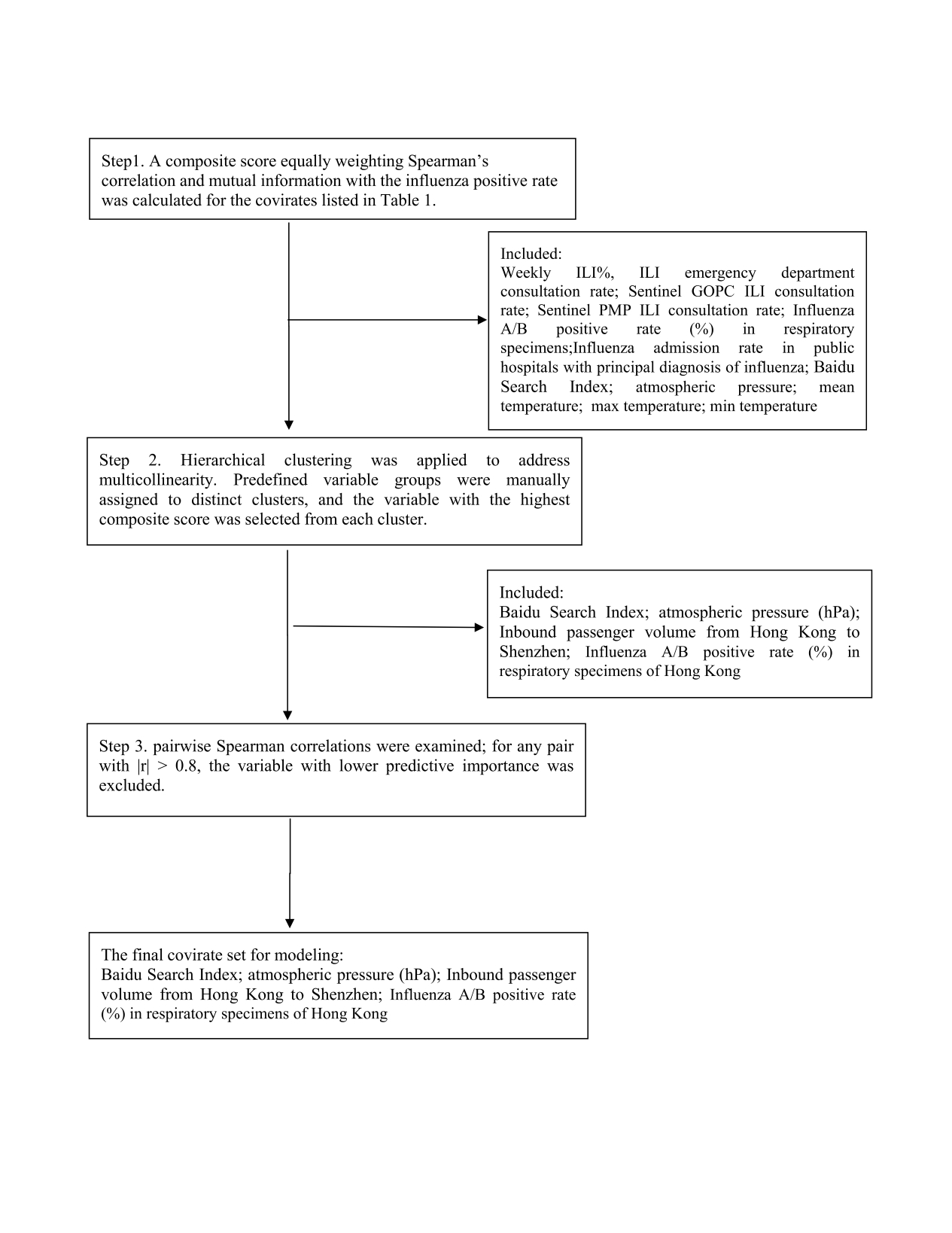

Supplement: SUPPLEMENTARY FIGURE 2 — The flow diagram of variable selection. [file Image_2.JPEG]

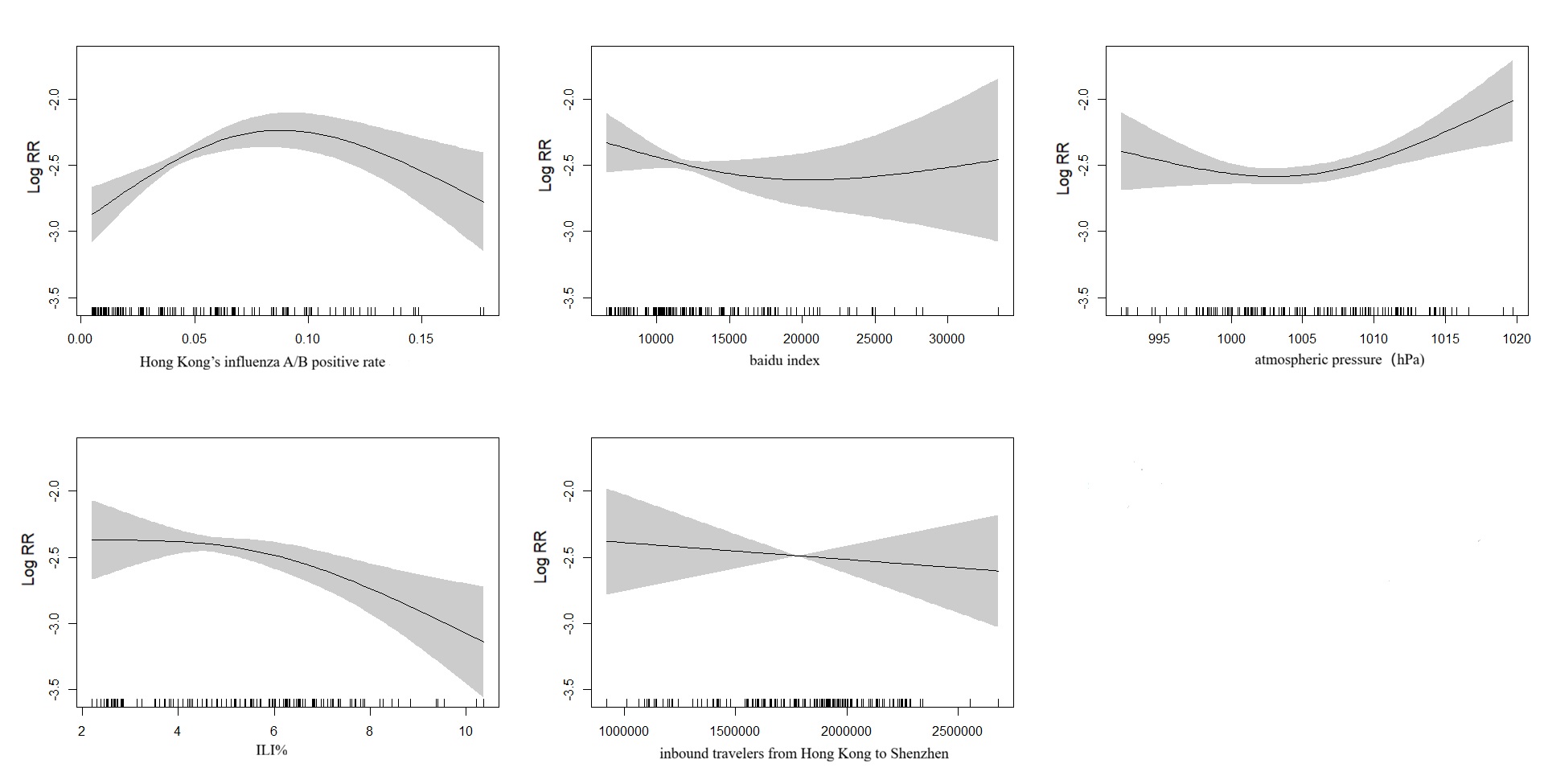

Supplement: SUPPLEMENTARY FIGURE 3 — Dose-response relationship curves of key preditors and influenza positive rate in Shenzhen. [file Image_3.JPEG]

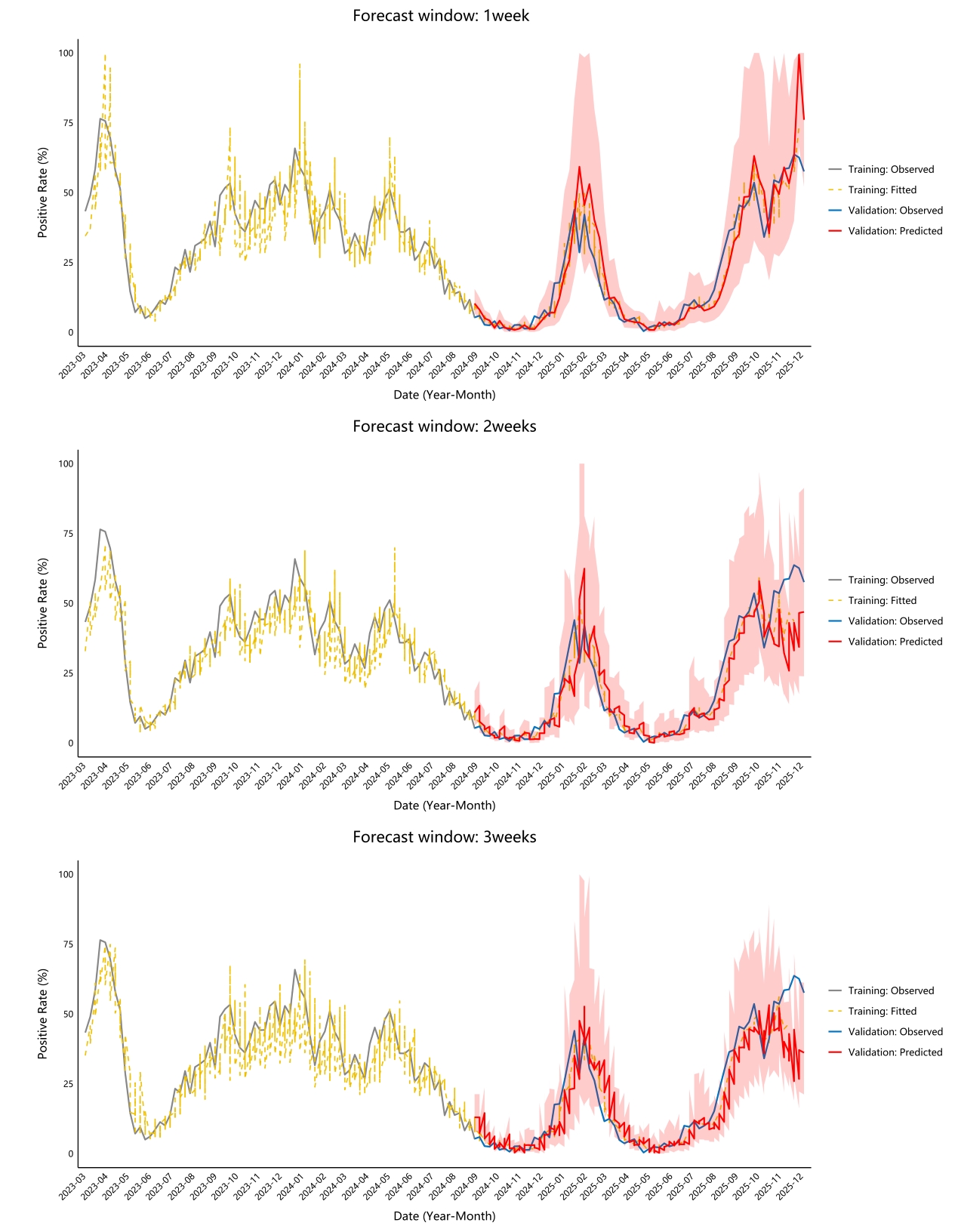

Supplement: SUPPLEMENTARY FIGURE 4 — The fitting and predictive performance of the SARIMAX model across different horizons. [file Image_4.JPEG]
